# Parametric magnon transduction to spin qubits

**DOI:** 10.1126/sciadv.adi2042

**Published:** 2024-03-20

**Authors:** Mauricio Bejarano, Francisco J. T. Goncalves, Toni Hache, Michael Hollenbach, Christopher Heins, Tobias Hula, Lukas Körber, Jakob Heinze, Yonder Berencén, Manfred Helm, Jürgen Fassbender, Georgy V. Astakhov, Helmut Schultheiss

**Affiliations:** ^1^ Helmholtz-Zentrum Dresden-Rossendorf, Institute for Ion Beam Physics and Materials Research, 01328 Dresden, Germany.; ^2^ Faculty of Electrical and Computer Engineering, Technical University of Dresden, 01062 Dresden, Germany.; ^3^ Max Planck Institute for Solid State Research, 70569 Stuttgart, Germany.; ^4^ Faculty of Physics, Technical University of Dresden, 01062 Dresden, Germany.; ^5^ Institute of Physics, Technical University of Chemnitz, 09107 Chemnitz, Germany.

## Abstract

The integration of heterogeneous modular units for building large-scale quantum networks requires engineering mechanisms that allow suitable transduction of quantum information. Magnon-based transducers are especially attractive due to their wide range of interactions and rich nonlinear dynamics, but most of the work to date has focused on linear magnon transduction in the traditional system composed of yttrium iron garnet and diamond, two materials with difficult integrability into wafer-scale quantum circuits. In this work, we present a different approach by using wafer-compatible materials to engineer a hybrid transducer that exploits magnon nonlinearities in a magnetic microdisc to address quantum spin defects in silicon carbide. The resulting interaction scheme points to the unique transduction behavior that can be obtained when complementing quantum systems with nonlinear magnonics.

## Teaser

Collective spin excitations in a microdisc offer unique nonlinearities for addressing spin-defect qubits.

## INTRODUCTION

The seminal proposal of quantum computers by R. Feynman in the 1980s (*1*) spurred a string of research and engineering developments in quantum computing hardware that spans from the first experimental realization of a quantum bit, or qubit, with trapped ions (*2*) to the latest 1121-superconducting qubit processor by IBM (*3*). Despite the remarkable progress in implementing qubits in a variety of hardware platforms such as superconducting circuits (*4*), trapped ions (*5*), defect centers (*6*, *7*), and semiconductor quantum dots (*8*), current error rates hamper a monolithic approach to building scalable quantum systems capable of processing, storage, and transmission of quantum information (*9*, *10*). This has motivated research into hybrid architectures for quantum information networks and leveraging the complementary advantages of modular building blocks based on distinct physical systems (*11*, *12*).

Under this heterogeneous approach, engineering interfaces that allow tunable transduction between the distinct physical components become increasingly important. So far, research efforts have explored realizing these interfaces via magnetic fields, phonons (the quanta of lattice vibrations), and, to a lesser extent, magnons (the quanta of collective spin excitations in magnetic materials) (*9*, *13*). Magnon-based transducers, despite being comparatively unexplored in the field, are especially promising for quantum computing due to the unique functionalities provided by their wide range of magnon interactions and intrinsic nonlinear phenomena (*14*–*16*). Nevertheless, most work in quantum magnonics has focused instead on linear magnon dynamics to mediate the interaction between microwave photons and superconducting or spin defect qubits (*14*–*24*). Furthermore, the vast majority of the reported hybrid systems are composed of yttrium iron garnet (YIG) and nitrogen-vacancy (NV) defects in diamond, both of which present fabrication challenges for wafer-scale integration (*14*, *15*, *25*). The introduction of magnon nonlinearities into the quantum architecture, which could lead to enhanced transduction behavior and tunability, has remained largely unexplored.

In this work, we fill this gap by presenting a quantum transducer that exploits magnon nonlinearities in a ferromagnetic microdisc to mediate the microwave interaction with spin qubits in silicon carbide (SiC). Our hybrid system harnesses parametric magnon effects to downconvert the microwave driving frequency and address off-resonant ensembles of spin qubits. This indirect scheme minimizes the microwave footprint by using highly-confined magnon stray fields to drive the spin qubits at room temperature. We envision our results as a proof of principle of a wafer-compatible hybrid quantum system that could help expand the quantum engineer’s toolbox by enhancing quantum systems with the rich nonlinear physics of magnons.

## RESULTS

### Hybrid nonlinear magnon-quantum system

**Fig. 1.**
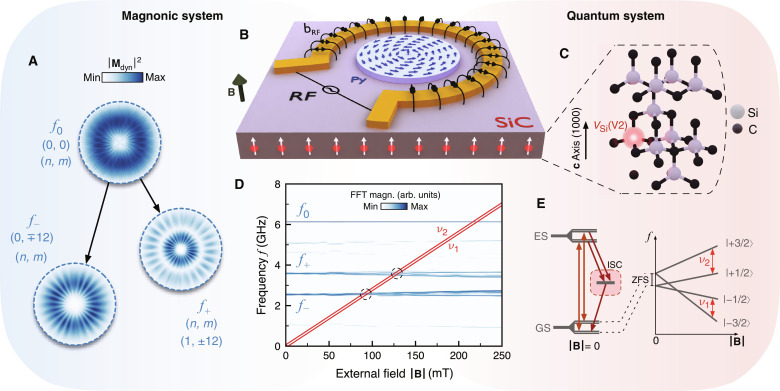
Parametric quantum transducer based on vortex magnons. (**A**) Parametric generation of magnons via three-magnon splitting. The initial pump mode *f*_0_ splits into secondary modes *f*_+_ and *f*_−_ following specific selection rules. (*n*, *m*) are the radial and azimuthal indices of the quantized vortex magnons. **M**_dyn_ denotes the dynamic component of the magnetization vector. (**B**) A permalloy (Py) disc with 5.1-μm diameter and 50-nm thickness on top of a SiC substrate hosting an ensemble of *V*_Si_(V2) defects. An on-chip antenna surrounding the disc is used to excite the vortex magnons. (**C**) Crystallographic structure of the 4H SiC polytype showing the *V*_Si_(V2) defect residing at the quasicubic lattice site. (**D**) General coupling principle. The ν_1,2_ resonances of the *V*_Si_(V2) cross the secondary magnon modes at two distinct field ranges (dashed circles) allowing pure-magnon addressability of the spin qubits. The observed branching of the secondary *f*_+_ and *f*_−_ modes with increasing out-of-plane field ∣**B**∣ corresponds to the exchange-induced splitting of the degenerate doublets as the vortex transitions into a vortex-cone state (*29*, *64*). (**E**) Ground state energy level structure and optical transitions of *V*_Si_(V2). Intersystem crossing (ISC) allows spin initialization and readout. At ∣**B**∣ ≠ 0, energy levels split due to Zeeman interaction. ZFS, zero-field splitting. The spatial profiles of the modes in (A) and their FFT (fast Fourier transform) spectra in (D) were obtained using micromagnetic simulations.

Our proposed hybrid system, shown in Fig. 1, is composed of two main elements: a nonlinear magnonic system as the transducing component and an ensemble of silicon-vacancy (*V*_Si_) defects in SiC as the quantum component. The magnonic system is a 50-nm-thick permalloy (Ni_81_Fe_19_) disc with a 5.1-μm diameter in a flux-closure vortex state (Fig. 1B) (*26*). The magnon spectrum of this system is quantized into degenerate doublet states described by their indices (*n*, *m*) with *n* = 0,1,2, … the number of nodes in the radial direction and *m* = 0, ±1, ±2, … the number of periods in the azimuthal direction (fig. S3) (*27*–*30*). Examples of these vortex eigenmodes are shown in Fig. 1A. We use an on-chip omega-shaped antenna that surrounds the disc to inductively couple to these vortex modes (see Fig. 1B). Because of the rotational symmetry of this excitation field, only modes with *m* = 0 are efficiently excited by the antenna.

The quantum component (Fig. 1, C and E) is a 200-nm-deep *V*_Si_-rich layer in the 4H SiC polytype substrate (fig. S1). The electronic spin state associated with this defect center has been extensively explored as an atomic-scale spin qubit with long coherence times even at room temperature (*31*, *32*). Figure 1E shows the energy levels and optical transitions of the *V*_Si_ occupying the quasicubic lattice site, referred to as *V*_Si_(V2) in the literature (*33*), which is the one we focus on in this work. The energy levels of this defect are quadruplets with *S* = 3/2, split at both ground and excited states into *m_s_* = ±1/2, ±3/2 (*31*, *34*–*36*). A spin-dependent intersystem crossing enables spin initialization and readout via photoluminescence (PL). At zero magnetic field, the energy levels are spin-split by the zero-field splitting of 2D = 70 MHz, where *D* is the crystal field constant. At ∣**B**∣ ≫ 2.5 mT, the *V*_Si_(V2) spin resonance frequencies ν_1,2_ shift linearly with ∣**B**∣ due to Zeeman splitting following

$$\nu_{1,2}=\nu_{0}\pm D\left(3\text{co}s^{2}\theta -1\right)$$ (1)

with ν_0_ = γ∣**B**∣, ∣γ∣ = 28 MHz/mT being the electron gyromagnetic ratio, and θ being the angle between the magnetic field and the **c **axis (*31*, *34*, *37*).

At the core of this hybrid system is a “three-wave mixing” process involving three vortex modes: an initial pump mode *f*_0_ with *m* = 0 and two secondary modes *f*_+_ and *f*_−_ with *m* ≠ 0. Above a certain microwave power threshold the pump mode *f*_0_ becomes unstable and spontaneously splits into the two secondary modes (*38*, *39*). The selection rules of this three-magnon splitting (3MS) process require the secondary magnon modes to have opposite azimuthal index *m*, but different radial indices *n* which, in return, leads to distinct frequencies *f*_−_ = *f*_0_/2 − Δ*f* and *f*_+_ = *f*_0_/2 + Δ*f* (*40*, *41*). For the particular case of the 5.1-μm-diameter vortex disc, it was shown that high-power microwave excitation at *f*_exc_ = 6.1 GHz results in the spontaneous splitting of the directly-excited pump mode with indices (0, 0) into the doublets (0, ∓12) at f = 2.55 GHz and (1, ±12) at f = 3.55 GHz (see Fig. 1A) (*30*). Notably, as the vortex transitions into a vortex-cone state with increasing out-of-plane fields ∣**B**∣, the secondary modes show minimal change, facilitating frequency crossing points with the electron spin resonance of *V*_Si_(V2). At these crossings, the *V*_Si_(V2) are degenerate only to the secondary modes, leaving the pump mode *f*_0_ and the antenna microwaves off-resonant. Figure 1D summarizes this indirect coupling scheme between the secondary parametric magnon modes and the *V*_Si_(V2).

A remarkable consequence of our coupling mechanism is that the interaction between the magnons and the *V*_Si_(V2) is unidirectional. While 3MS is itself a reversible process [three-magnon confluence (*41*, *42*)], the generation of the initial pump mode *f*_0_ by the *V*_Si_(V2) spins is not possible as it would require simultaneous excitation of the secondary modes which, in turn, lie at different frequencies *f*_−_ and *f*_+_.

### Magnon-driven *V*_Si_ spins

**Fig. 2.**
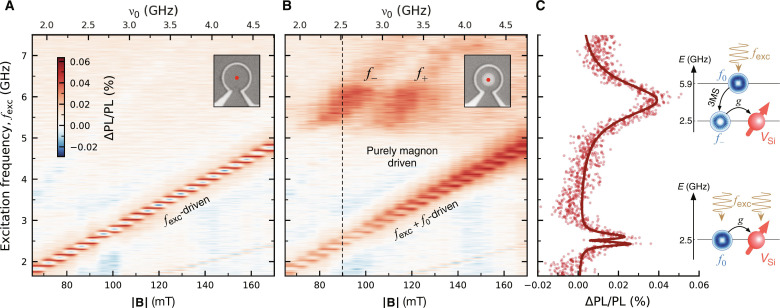
Optical detection of magnon-driven *V*_Si_(V2) spin transitions. (**A**) Reference ODMR spectra as a function of the external magnetic field ∣**B**∣ measured at the center of a microwave antenna without the vortex disc (see inset). The diagonal ΔPL/PL intensity corresponds to the field-dependent *V*_Si_(V2) resonances. The discrete-like resonances are due to coarse increments in the applied magnetic field (see the Supplementary Materials). (**B**) ODMR spectra as a function of external magnetic field ∣**B**∣ for *V*_Si_(V2) ensembles below the vortex disc (see inset). The off-diagonal signal around *f*_exc_ ∼ 6-GHz results from *V*_Si_(V2) spin transitions purely driven by the parametric magnon modes *f*_−_ and *f*_+_. (**C**) ODMR spectrum at ∣**B**∣ = 90-mT, as extracted along the dashed line in (B). The energy diagram illustrates the origin of the two distinct features in the spectrum. *g* denotes the spin-magnon coupling strength. The spectra in (A) and (B) were both obtained by applying 9 dBm of microwave power. 3MS, three-magnon splitting.

We start by experimentally demonstrating the driving of *V*_Si_(V2) spins by the parametric magnons in the vortex disc. Given the magnon-driven regime occurs at the two crossing points between the *V*_Si_(V2) resonances and the *f*_−_ and *f*_+_ modes (dashed circles in Fig. 1D), we use the magnetic field as an independent parameter to tune the *V*_Si_(V2) into and out of resonance with these modes while tracking the PL from the defects through optically detected magnetic resonance (ODMR) at room temperature. As a reference experiment, we first exclude the influence of magnons on the ODMR spectra by collecting the PL of *V*_Si_(V2) located at the center of an empty antenna, i.e., without the vortex disc. The resulting ODMR spectra, shown in Fig. 2A, display the expected linear field dependence of the *V*_Si_(V2) spin resonances as previously described by Eq. 1 for θ = 0, i.e., with out-of-plane magnetic fields.

The ODMR spectra of *V*_Si_(V2) defects below the vortex disc are shown in Fig. 2B. Here, we distinguish two distinct resonances of different origins: the resonances increasing linearly with the field and the off-diagonal resonances around *f*_exc_ ∼ 6 GHz. The linear resonances show similarities with the antenna-driven resonances in Fig. 2A, however, are a result of the combined action of the microwave antenna fields and the dynamic stray fields from the linear vortex dynamics. Given that the microwave power delivered by the antenna is kept constant, the increase of the ODMR intensity along the diagonal is an indication of the concomitant increase of the dynamic stray fields, which together result in overall larger microwave driving fields. This increase in dynamic stray fields occurs because the vortex magnetization is more resonantly excited when the excitation frequency *f*_exc_ approaches the resonant frequency of the pump mode (fig. S4).

In contrast, the off-diagonal resonances around ∣**B**∣ = 90 mT and ∣**B**∣ = 115 mT in Fig. 2B are a result of a purely-magnonic driving of the *V*_Si_(V2) spin transitions. To illustrate this, in Fig. 2C, we show the ODMR spectrum at ∣**B**∣ = 90 mT as extracted along the dashed line in Fig. 2B. At this field, the *V*_Si_(V2) resonances are centered around ν_0_ ∼ 2.5 GHz (see second *x* axis on top of Fig. 2B). The apparent contradicting presence of ODMR intensities at *f*_exc_ ∼ 6 GHz suggests a microwave downconversion to the *V*_Si_(V2) resonance frequencies. We attribute this downconversion to the parametric generation of secondary magnons in the disc via the 3MS process detailed in the previous section. In good agreement with the simulation data shown in Fig. 1D, the parametric magnon modes, with frequencies *f*_−_ and *f*_+_, are resonant to the spin defects at ∣**B**∣ = 90 mT and ∣**B**∣ = 115 mT, respectively, and couple to the *V*_Si_(V2) with a spin-magnon coupling strength *g*. The absence of a level anti-crossing around *f*_exc_ ∼ 6 GHz reveals that the intrinsic decoherence mechanisms from the magnons, κ*_m_*, and from the *V*_Si_ ensembles, κ*_q_*, overcome the coupling strength, κ*_m_*, κ*_q_* > *g*, putting the system in the weak coupling regime. This regime is defined by cooperativities *C* < 1, with $C\equiv \frac{4g^{2}}{\kappa_{m}\kappa_{q}}$.

The large ODMR linewidth observed at *f*_exc_ ∼ 6 GHz in Fig. 2C can have multiple causes. On one hand, the 9 dBm of microwave power used to drive the 3MS is sufficiently high to broaden the ODMR linewidth and distort its lineshape (fig. S6). On the other hand, the counter-propagating secondary modes can induce Doppler broadening, as has been observed with whispering gallery modes coupled to atoms (*43*). In addition, inhomogeneous broadening caused by the interaction with the thermal-magnon bath could also play an important role.

### Threshold activation of magnon-driven *V*_Si_

**Fig. 3.**
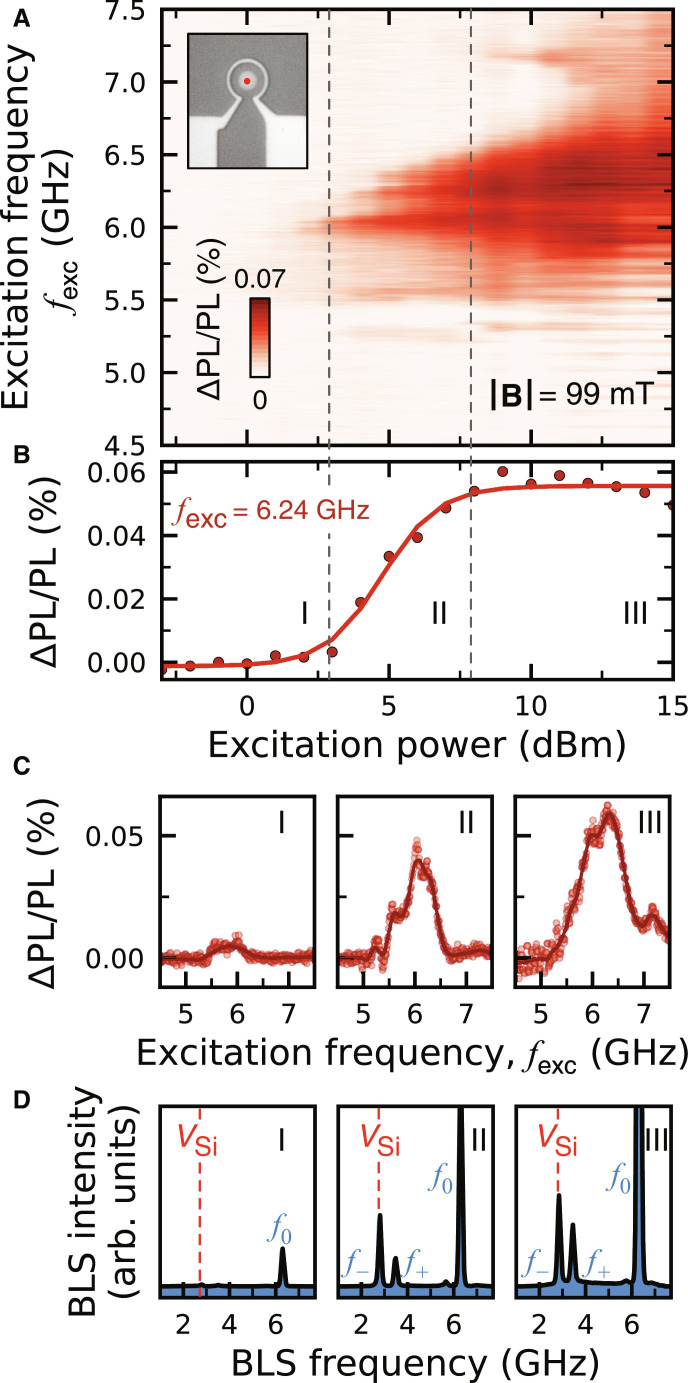
Threshold process of the parametric driving scheme. (**A**) ODMR spectra for increasing microwave excitation powers at ∣**B**∣ = 99-mT for *V*_Si_(V2) below the disc (see inset). (**B**) ODMR contrast averaged over a 20-MHz-frequency window centered at *f*_exc_= 6.24-GHz showing the characteristic threshold behavior of the three-magnon splitting. Three distinct microwave power ranges can be identified as follows: (I) below threshold, (II) above threshold, and (III) saturation. For each range, the average ODMR spectrum is shown in (**C**) and the corresponding average BLS spectrum is shown in (**D**). *V*_Si_ in (D) refers to ν_0_. Solid line in (B) is a sigmoidal fit to the data.

Now, we demonstrate the threshold activation of the *V*_Si_(V2) spins driven by the parametric magnons in the vortex disc. We complement ODMR measurements on *V*_Si_(V2) with micro-focused Brillouin light scattering microscopy (μBLS) on the disc to elucidate the concurrent magnon dynamics driving the changes in the PL of *V*_Si_(V2) centers. Figure 3A shows a power-dependent ODMR spectrum at ∣**B**∣ = 99 mT, where the *f*_−_ magnon mode drives the *V*_Si_(V2) spin transitions (as seen in Fig. 2B). While typical ODMR spectra are continuous with increasing microwave driving power (fig. S6), the measured ODMR spectra depict a threshold-like power dependence. This nonlinear onset of ODMR intensity at the off-resonant *f*_exc_ ∼ 6.1 GHz suggests that the appearance of the resonant magnon *f*_−_ occurs after the microwave power exceeds a critical threshold amplitude. This critical amplitude is associated with the instability threshold of the pump mode *f*_0_ as discussed before.

To further clarify the evolution of this nonlinear process, we extract the average ODMR contrast over a 20-MHz-frequency window centered at *f*_exc_ = 6.24 GHz and identify three separate power ranges: (I) below threshold, (II) above threshold, and (III) saturation (Fig. 3B). For each range, Fig. 3 (C and D) shows the average ΔPL/PL of the *V*_Si_(V2) spins and the average μBLS spectrum from the disc, respectively. Below threshold (I), there is no change in the PL from the *V*_Si_(V2) spins as there is no magnon mode at the *V*_Si_(V2) resonance frequencies. In this regime, only the pump mode *f*_0_, which is nonresonant to the *V*_Si_(V2) spins, is present. Above threshold (II), there is a nonzero μBLS signal intensity at the resonance frequency of the secondary magnon *f*_−_ (Fig. 3D). As the magnon mode *f*_−_ matches the *V*_Si_(V2) resonance frequencies, it drives *V*_Si_(V2) spin transitions which induces a change in PL. At saturation (III), the intensity of the secondary mode *f*_−_ is the largest, which gives rise to the high change in PL observed in Fig. 3C. By further increasing the excitation power, higher-order interaction processes between the secondary magnons, such as four-magnon scattering, become relevant. Consequently, this leads to decoherence, limiting the energy flux between the pump and secondary magnons (*30*, *38*) and resulting in the observed saturation in ΔPL/PL.

### Room temperature spin-magnon coupling

**Fig. 4.**
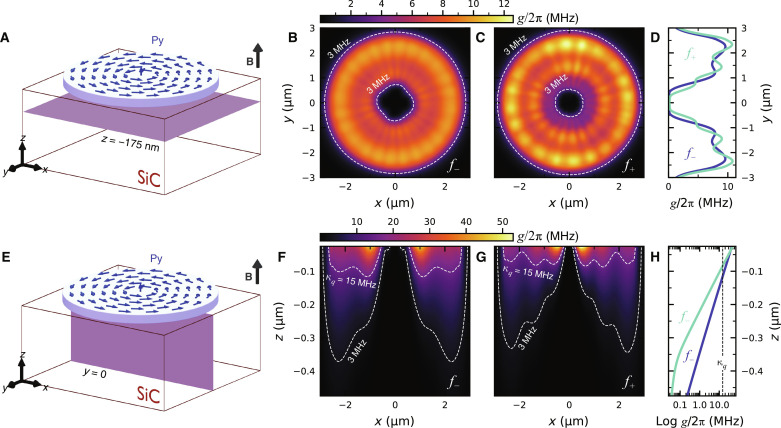
Room-temperature coupling between the vortex magnons and the *V*_Si_(V2) spins. (**A**) Schematic depicting plane located 175-nm below the bottom surface of the disc, where the spin-magnon coupling strength is calculated. (**B** and **C**) Coupling strength *g* for the parametric modes *f*_−_ and *f*_+_ at ∣**B**∣ = 90-mT and ∣**B**∣ = 130-mT, respectively, at the plane shown in (A). (**D**) Coupling strength extracted along *x *= 0-μm from the intensity maps shown in (B) and (C). (**E**) Schematic depicting cross-sectional plane located at *y *= 0-μm starting 25-nm below the disc. (**F** and **G**) Coupling strength *g* for the parametric magnons *f*_−_ and *f*_+_ at ∣**B**∣ = 90-mT and ∣**B**∣ = 130-mT, respectively, at the plane shown in (E). (**H**) Coupling strength for the *f*_−_ and *f*_+_ modes along x = −1.05-μm and *x *= 0.6-μm as extracted from (F) and (G), respectively.

Having experimentally demonstrated the working principle of our hybrid transducer, we now characterize the room temperature spin-magnon coupling between the secondary vortex modes and the *V*_Si_(V2) spins. We build upon the theoretical work of Candido *et al*. (*44*) and define the spin-magnon coupling strength *g* as follows

$$g\left(r_{V_{\text{Si}}}\right)=\frac{\sqrt{2}}{4}\gamma \mu_{0}h_{⊥}^{\text{rf}}\left(r_{V_{\text{Si}}}\right)$$ (2)

with μ_0_ being the vacuum permeability, $h_{⊥}^{\text{rf}}$ being the magnon stray fields perpendicular to the *V*_Si_(V2) spin axis, and **r**_*V*_Si__ = (*x*, *y*, *z*) being the coordinates of the *V*_Si_(V2) defect. Using a combination of micromagnetic simulations and analytical calculations (see the Supplementary Materials), we obtain the magnon stray fields $h_{⊥}^{\text{rf}}$ and use Eq. 2 to derive the spatial profile of the spin-magnon coupling strength *g* below the vortex disc. Figure 4 (B and C) shows the calculated coupling strength for the secondary magnon modes *f*_−_ and *f*_+_ at a plane 175 nm below the disc (depicted in Fig. 4A), which lies within the *V*_Si_(V2)-rich layer in our sample (fig. S1). Figure 4D shows the variation of the coupling strength in Fig. 4 (B and C) along a line at *x *= 0 μm. Because of the high spin density of permalloy, which results in a high saturation magnetization μ_0_*M*_S,Py _= 1 T (*45*, *46*), we obtain coupling strengths as large as 10-MHz. For comparison purposes, the coupling strengths of other reported NV/YIG hybrid systems is around 1-MHz for spin-magnon distances in the order of tens of nanometers (*47*, *48*). These lower coupling strengths are a result of the much lower spin density of YIG, which yields a smaller saturation magnetization μ_0_*M*_S,YIG_ = 0.170 T (*49*).

We now show the coupling strength for the *f*_+_ and *f*_−_ modes across the thickness of the SiC substrate in Fig. 4 (F and G) (see cross-sectional plane in Fig. 4E) and compare it with the intrinsic decoherence rates of the system. We estimate κ*_q_* = 2π× 15 MHz for the spin ensemble, and κ_*m*,−_ = 2π× 89 MHz and κ_*m*,+_ = 2π× 73 MHz for the damping rates of the *f*_−_ and *f*_+_ modes, correspondingly (see the Supplementary Materials). Here, it is evident that the spin-magnon coupling strengths at the depth of the *V*_Si_-rich layer are smaller than the decoherence rates from the magnons and from the spin ensemble, confirming that the system is in the weak coupling regime. Nonetheless, we can also observe an important consequence of the pure magnon-spin coupling mechanism. As the antenna microwaves are off-resonant to the spin defects, the microwave driving fields are of purely magnonic origin. These magnon stray fields decay quickly in space with a 1/*r*^3^ dependence, confining the microwave control to a submicrometer volume below the disc. This is in stark contrast to the ∼1-mm^3^-sized volumes typically achieved with transmission line microwave antennas (*9*). As shown in Fig. 4 (F and G), the magnon stray fields result in *g*/2π > 15 MHz confined to the top ∼100 nm close to the disc. To expand on this microwave confinement, in Fig. 4H, we plot the spatial decay of the coupling strength at *x* = –1.05 μm (for *f*_−_; Fig. 4F) and at *x* = 0.6 μm (for *f*_+_; Fig. 4G). Although spin defects at *z* = –175 nm (like the defects in our sample) experience coupling strengths in the range of 5 to 7 MHz, there is a broad depth-range to engineer defects with stronger coupling. Precise control of the implantation energies allows the creation of defects closer to the surface to achieve large spin-magnon coupling strengths but still deep enough to avoid detrimental surface effects (*50*).

## DISCUSSION

In summary, the hybrid quantum transducer presented in this work exploits magnon nonlinearities to enhance microwave transduction to spin qubits. The resulting spin-magnon coupling scheme points to a transduction behavior different from the one observed in hybrid systems with linearly-excited magnons. Whereas the dipolar coupling between the spin centers and the linear magnons is always “on,” the parametric processes at the core of our hybrid system represent a way to selectively tune the spin-magnon coupling “on” and “off”. With such control of the coupling, it would be possible to protect the spin centers against any resonant magnon noise-induced decoherence. Moreover, given the nature of the resulting coupling is purely of magnonic origin, the microwave driving fields are confined to submicrometer volumes, in stark contrast with the microwave fields from antennas, which typically extend over hundreds of micrometers on the sample plane (*51*). Looking forward, this could be an interesting path toward reducing the spillover cross-talk (*52*) as the number of qubits scales in future quantum chips.

Our proposed system represents a departure from the traditional NV/YIG composition by using permalloy and silicon vacancies in silicon carbide, two materials highly compatible with industry-standard fabrication protocols and easier integrability into heterogeneous quantum architectures (*53*–*55*). Despite the large spin-magnon coupling strengths calculated, our system is not in the strong coupling regime, due to the large damping rates from the vortex magnons and the *V*_Si_ spin ensemble. We expect that coupling to single spin defects instead of spin ensembles will result in *C* > 1 (fig. S7), therefore bringing the system into the strong coupling regime. Moreover, by going to cryogenic temperatures, the damping processes intrinsic to metallic ferromagnetic thin films should be reduced (*56*). Another interesting perspective is exploiting the nonlinear 3MS process to generate squeezed magnon states (*57*–*59*). Such nonclassical states have the potential to exponentially enhance the coupling strengths and cooperativities in hybrid quantum systems (*60*, *61*).

Quantum magnonics focused thus far on coupling long-range magnon propagations to mediate microwave interactions with spin qubits, naturally directing research efforts into YIG-based hybrid systems due to its low damping coefficient. This, however, left a large body of magnon phenomena unexplored. By introducing a nonlinear magnonic system, we provide alternative perspectives for engineering quantum interfaces to spin qubits and motivate further research into uncovering the interesting phenomena lying at the intersection of nonlinear magnonics and quantum systems.

## MATERIALS AND METHODS

### Sample fabrication

The 10-mm × 10-mm sample was diced from a high-purity semi-insulating 4H-SiC wafer with a crystal axis along the surface normal. To create the *V*_Si_ defects, the entire sample was irradiated with protons at a fluence of 1 × 10^15^ cm^−2^ with an energy of 30 keV. This procedure yields a theoretical penetration depth of the protons around 200 nm, which was obtained with Monte Carlo simulations in the software SRIM (fig. S1). After irradiation, electron beam lithography, sputter deposition, and subsequent liftoff were performed to fabricate the Ni_81_Fe_19_ disc (5.1-μm diameter and 50-nm thick) and the on-chip antenna (6 nm Cr/150 nm Au).

### Experimental setups

To measure ODMR, a custom-built confocal scanning microscope was used (fig. S2). The *V*_Si_ defects were optically excited with a 785-nm continuous-wave laser diode (Thorlabs LP785-SF100) which is focused onto the sample by a microscope objective (Zeiss, LD Plan Neofluar 63×/NA 0.75). This objective has an adjustable coverglass correction to compensate for the aberrations introduced by illuminating the *V*_Si_ defects through the SiC substrate. The emitted PL, with a spectrum centered at 900-nm, is collected through the same objective and then filtered by appropriate dichroic mirrors and longpass filters before finally being measured by a silicon avalanche photodetector (Thorlabs, APD440A).

A dedicated μBLS microscope was used to detect the magnon dynamics within the permalloy disc. μBLS uses the inelastic scattering of photons with magnons to perform a space- and frequency-resolved detection of the magnon intensity (*62*). For this purpose, a continuous-wave 532-nm solid-state laser (Novanta Photonics, Torus 532) is focused onto the sample using a microscope objective (Zeiss, LD EC Epiplan Neofluar 100×/NA 0.75) yielding a spot size of approximately 320 nm. The scattered light is collected by the same objective and analyzed with a high-resolution tandem Fabry-Perot interferometer (JRS Scientific Instruments, TFP-2).

To excite the magnetization dynamics in the permalloy disc and drive spin transitions in the *V*_Si_ defects, microwaves from a signal generator (Keysight, 5183B) were fed to the on-chip antenna through nonmagnetic RF probes (GGB Industries Inc., Model 40A), which are positioned onto the sample using *xyz* micro-positioning stages.

For the ODMR measurements, the sample was placed on a sample holder that allowed for laser illumination via the backside of the SiC substrate, to enable probing of the *V*_Si_ defects directly below the permalloy disc. The ΔPL/PL signal was obtained using a lock-in detection scheme, with the photovoltage from the photodiode sampled by a lock-in amplifier (Signal Recovery 7265) locked to a reference sinusoidal signal which modulates the amplitude of the microwaves being fed to the sample.

For both confocal and μBLS microscopes, a permanent magnet on a motorized stage is used to deliver out-of-plane magnetic fields along the crystal axis of the SiC substrate. All the experiments in this work were performed at room temperature.

### Micromagnetic simulations

The micromagnetic simulations for Fig. 1 (A and D) were obtained with MuMax3 (*63*). The disc was discretized into 512 × 512 × 1 rectangular cells, with lengths Δx = 10 nm, Δy = 10 nm, and Δz = 50 nm. The standard material parameters for permalloy were used: saturation magnetization M_S_ = 810 kA/m, exchange stiffness *A_ex_* = 13 pJ/mm, Gilbert damping parameter α_G _= 0.007, and gyromagnetic ratio γ = 29.6 GHz/T. The equilibrium configuration was set to be a vortex state with chirality *c* = −1 and polarity *p* = 1.

The magnetization dynamics were excited by a spatially homogeneous time-varying sinusoidal magnetic field of 5-mT amplitude and 6.1 GHz in the out-of-plane direction. The time evolution of the magnetization was recorded every 10 ps for 100 ns, for a total of 10,000 samples. To obtain the power spectrum of the modes in the disc, a fast Fourier transform (FFT) in time for each cell was calculated and the resulting spectrum was spatially averaged over the whole disc. The spatial mode profiles in Fig. 1A were obtained by performing a backward windowed FFT around the frequency of interest.

### Calculation of the magnon stray fields

To calculate the magnon magnetic fields, we consider the *N* cells of the discretized disc as *N* point dipoles located at the center of each cell and calculate the total dipolar magnetic field at an arbitrary point *P* in space, as the resulting collective contribution from each *i*th cell

$$\mu_{0}H\left(m,r\right)=\sum_{i}^{N}‍\frac{\mu_{0}}{4\pi }\frac{3\left(m_{i}\cdot {\hat{r}}_{i}\right){\hat{r}}_{i}-m_{i}}{r_{i}^{3}}$$ (3)

with **m***_i_* being the vector dipole moment of the *i*th cell and ${\hat{r}}_{i}$ being the normalized distance vector from the *i*th cell to point *P*. By repeating this algorithm for all the time samples of the micromagnetic simulation, we obtain the time-dependent magnetic field μ_0_*H*(*t*, **m**, **r**) at point *P*. We use this algorithm for calculating the dipolar magnetic fields at the evaluation planes shown in Fig. 4. The resulting fields will contain the contributions from the overall magnetization dynamics in the disc. To isolate the contribution from a specific vortex magnon, *f*_+_ or *f*_−_, we perform an FFT in time for each evaluation point and then a backward windowed FFT around the frequency *f*_+_ or *f*_−_. The magnetic fields used in Eq. 2 are the transversal components of these reconstructed magnon magnetic fields.

## Supplementary Materials

### This PDF file includes:

Sections S1 to S5 Figs. S1 to S7 References

## References

[1] R. P. Feynman, Simulating physics with computers. Int. J. Theor. Phys. 21, 467–488 (1982).

[2] J. I. Cirac, P. Zoller, Quantum computations with cold trapped ions. Phys. Rev. Lett. 74, 4091–4094 (1995). 10058410 10.1103/PhysRevLett.74.4091

[3] D. Castelvecchi, IBM releases first-ever 1,000-qubit quantum chip. Nature 624, 238–238 (2023). 38053007 10.1038/d41586-023-03854-1

[4] Y. Nakamura, Y. A. Pashkin, J. S. Tsai, Coherent control of macroscopic quantum states in a single-Cooper-pair box. Nature 398, 786–788 (1999).

[5] C. D. Bruzewicz, J. Chiaverini, R. McConnell, J. M. Sage, Trapped-ion quantum computing: Progress and challenges. Appl. Phys. Rev. 6, 021314 (2019).

[6] F. Jelezko, J. Wrachtrup, Single defect centres in diamond: A review. Phys. Status Solidi A 203, 3207–3225 (2006).

[7] P. G. Baranov, A. P. Bundakova, A. A. Soltamova, S. B. Orlinskii, I. V. Borovykh, R. Zondervan, R. Verberk, J. Schmidt, Silicon vacancy in SiC as a promising quantum system for single-defect and single-photon spectroscopy. Phys. Rev. B 83, 125203 (2011).

[8] D. Loss, D. P. DiVincenzo, Quantum computation with quantum dots. Phys. Rev. A 57, 120–126 (1998).

[9] D. S. Wang, M. Haas, P. Narang, Quantum interfaces to the nanoscale. ACS Nano 15, 7879–7888 (2021). 33999597 10.1021/acsnano.1c01255

[10] S. Pirandola, S. L. Braunstein, Physics: Unite to build a quantum Internet. Nature 532, 169–171 (2016). 27075080 10.1038/532169a

[11] C. Monroe, R. Raussendorf, A. Ruthven, K. R. Brown, P. Maunz, L.-M. Duan, J. Kim, Large-scale modular quantum-computer architecture with atomic memory and photonic interconnects. Phys. Rev. A 89, 022317 (2014).

[12] Z.-L. Xiang, S. Ashhab, J. Q. You, F. Nori, Hybrid quantum circuits: Superconducting circuits interacting with other quantum systems. Rev. Mod. Phys. 85, 623–653 (2013).

[13] N. Lauk, N. Sinclair, S. Barzanjeh, J. P. Covey, M. Saffman, M. Spiropulu, C. Simon, Perspectives on quantum transduction. Quantum Sci. Technol. 5, 020501 (2020).

[14] D. D. Awschalom, C. R. Du, R. He, F. J. Heremans, A. Hoffmann, J. Hou, H. Kurebayashi, Y. Li, L. Liu, V. Novosad, J. Sklenar, S. E. Sullivan, D. Sun, H. Tang, V. Tyberkevych, C. Trevillian, A. W. Tsen, L. R. Weiss, W. Zhang, X. Zhang, L. Zhao, C. W. Zollitsch, Quantum engineering with hybrid magnonic systems and materials (*Invited Paper*). IEEE Trans. Quantum Eng. 2, 1–36 (2021).

[15] Y. Li, W. Zhang, V. Tyberkevych, W.-K. Kwok, A. Hoffmann, V. Novosad, Hybrid magnonics: Physics, circuits, and applications for coherent information processing. J. Appl. Phys. 128, 130902 (2020).

[16] D. Lachance-Quirion, Y. Tabuchi, A. Gloppe, K. Usami, Y. Nakamura, Hybrid quantum systems based on magnonics. Appl. Phys. Express 12, 070101 (2019).

[17] D. Lachance-Quirion, Y. Tabuchi, S. Ishino, A. Noguchi, T. Ishikawa, R. Yamazaki, Y. Nakamura, Resolving quanta of collective spin excitations in a millimeter-sized ferromagnet. Sci. Adv. 3, e1603150 (2017). 28695204 10.1126/sciadv.1603150 PMC5498106

[18] D. Lachance-Quirion, S. P. Wolski, Y. Tabuchi, S. Kono, K. Usami, Y. Nakamura, Entanglement-based single-shot detection of a single magnon with a superconducting qubit. Science 367, 425–428 (2020). 31974250 10.1126/science.aaz9236

[19] Y. Tabuchi, S. Ishino, A. Noguchi, T. Ishikawa, R. Yamazaki, K. Usami, Y. Nakamura, Coherent coupling between a ferromagnetic magnon and a superconducting qubit. Science 349, 405–408 (2015). 26160378 10.1126/science.aaa3693

[20] P. Andrich, C. F. de las Casas, X. Liu, H. L. Bretscher, J. R. Berman, F. J. Heremans, P. F. Nealey, D. D. Awschalom, Long-range spin wave mediated control of defect qubits in nanodiamonds. Npj Quantum Inf. 3, 28 (2017).

[21] T. van der Sar, F. Casola, R. Walsworth, A. Yacoby, Nanometre-scale probing of spin waves using single electron spins. Nat. Commun. 6, 7886 (2015). 26249673 10.1038/ncomms8886 PMC4918315

[22] F. Casola, T. van der Sar, A. Yacoby, Probing condensed matter physics with magnetometry based on nitrogen-vacancy centres in diamond. Nat. Rev. Mater. 3, 17088 (2018).

[23] M. Fukami, D. R. Candido, D. D. Awschalom, M. E. Flatté, Opportunities for long-range magnon-mediated entanglement of spin qubits via on- and off-resonant coupling. PRX Quantum 2, 040314 (2021).

[24] Y. Li, V. G. Yefremenko, M. Lisovenko, C. Trevillian, T. Polakovic, T. W. Cecil, P. S. Barry, J. Pearson, R. Divan, V. Tyberkevych, C. L. Chang, U. Welp, W.-K. Kwok, V. Novosad, Coherent coupling of two remote magnonic resonators mediated by superconducting circuits. Phys. Rev. Lett. 128, 047701 (2022). 35148146 10.1103/PhysRevLett.128.047701

[25] S. Kosen, A. F. van Loo, D. A. Bozhko, L. Mihalceanu, A. D. Karenowska, Microwave magnon damping in YIG films at millikelvin temperatures. APL Mater. 7, 101120 (2019).

[26] T. Shinjo, T. Okuno, R. Hassdorf, K. Shigeto, T. Ono, Magnetic vortex core observation in circular dots of permalloy. Science 289, 930–932 (2000). 10937991 10.1126/science.289.5481.930

[27] M. Buess, R. Höllinger, T. Haug, K. Perzlmaier, U. Krey, D. Pescia, M. R. Scheinfein, D. Weiss, C. H. Back, Fourier transform imaging of spin vortex eigenmodes. Phys. Rev. Lett. 93, 077207 (2004). 15324274 10.1103/PhysRevLett.93.077207

[28] M. Buess, T. P. J. Knowles, R. Höllinger, T. Haug, U. Krey, D. Weiss, D. Pescia, M. R. Scheinfein, C. H. Back, Excitations with negative dispersion in a spin vortex. Phys. Rev. B 71, 104415 (2005).

[29] B. Ivanov, C. Zaspel, High frequency modes in vortex-state nanomagnets. Phys. Rev. Lett. 94, 027205 (2005). 15698225 10.1103/PhysRevLett.94.027205

[30] K. Schultheiss, R. Verba, F. Wehrmann, K. Wagner, L. Körber, T. Hula, T. Hache, A. Kákay, A. A. Awad, V. Tiberkevich, A. N. Slavin, J. Fassbender, H. Schultheiss, Excitation of whispering gallery magnons in a magnetic vortex. Phys. Rev. Lett. 122, 097202 (2019). 30932517 10.1103/PhysRevLett.122.097202

[31] H. Kraus, V. A. Soltamov, D. Riedel, S. Väth, F. Fuchs, A. Sperlich, P. G. Baranov, V. Dyakonov, G. V. Astakhov, Room-temperature quantum microwave emitters based on spin defects in silicon carbide. Nat. Phys. 10, 157–162 (2014).

[32] M. Widmann, S.-Y. Lee, T. Rendler, N. T. Son, H. Fedder, S. Paik, L.-P. Yang, N. Zhao, S. Yang, I. Booker, A. Denisenko, M. Jamali, S. A. Momenzadeh, I. Gerhardt, T. Ohshima, A. Gali, E. Janzén, J. Wrachtrup, Coherent control of single spins in silicon carbide at room temperature. Nat. Mater. 14, 164–168 (2015). 25437256 10.1038/nmat4145

[33] E. Sörman, N. T. Son, W. M. Chen, O. Kordina, C. Hallin, E. Janzén, Silicon vacancy related defect in 4H and 6H SiC. Phys. Rev. B 61, 2613–2620 (2000).

[34] E. Janzén, A. Gali, P. Carlsson, A. Gällström, B. Magnusson, N. T. Son, The silicon vacancy in SiC. Phys. B Condens. Matter 404, 4354–4358 (2009).

[35] N. Mizuochi, S. Yamasaki, H. Takizawa, N. Morishita, T. Ohshima, H. Itoh, J. Isoya, Continuous-wave and pulsed EPR study of the negatively charged silicon vacancy with S = 3/2 and C_3v_ symmetry in n -type 4*H* − SiC. Phys. Rev. B 66, 235202 (2002).

[36] S. A. Tarasenko, A. V. Poshakinskiy, D. Simin, V. A. Soltamov, E. N. Mokhov, P. G. Baranov, V. Dyakonov, G. V. Astakhov, Spin and optical properties of silicon vacancies in silicon carbide—A review. Phys. Status Solidi B 255, 1700258 (2018).

[37] D. Simin, F. Fuchs, H. Kraus, A. Sperlich, P. G. Baranov, G. V. Astakhov, V. Dyakonov, High-precision angle-resolved magnetometry with uniaxial quantum centers in silicon carbide. Phys. Rev. Applied 4, 014009 (2015).

[38] V. S. L’vov, Nonlinear dynamics and kinetics of magnons, in *Nonlinear Waves 3*, A. V. Gaponov-Grekhov, M. I. Rabinovich, J. Engelbrecht, Eds. (Springer Berlin Heidelberg, 1990), pp. 224–239.

[39] H. Suhl, The theory of ferromagnetic resonance at high signal powers. J. Phys. Chem. Solid 1, 209–227 (1957).

[40] R. E. Camley, Three-magnon processes in magnetic nanoelements: Quantization and localized mode effects. Phys. Rev. B 89, 214402 (2014).

[41] R. Verba, L. Körber, K. Schultheiss, H. Schultheiss, V. Tiberkevich, A. Slavin, Theory of three-magnon interaction in a vortex-state magnetic nanodot. Phys. Rev. B 103, 014413 (2021).

[42] V. L’vov, *Wave Turbulence under Parametric Excitation: Applications to Magnets*, Springer Series in Nonlinear Dynamics (Springer Berlin Heidelberg, 2012).

[43] D. W. Vernooy, A. Furusawa, N. P. Georgiades, V. S. Ilchenko, H. J. Kimble, Cavity QED with high-*Q* whispering gallery modes. Phys. Rev. A 57, R2293–R2296 (1998).

[44] D. R. Candido, G. D. Fuchs, E. Johnston-Halperin, M. E. Flatté, Predicted strong coupling of solid-state spins via a single magnon mode. Mater. Quantum Technol. 1, 011001 (2021).

[45] K. Ounadjela, H. Lefakis, V. S. Speriosu, C. Hwang, P. S. Alexopoulos, Thickness dependence of magnetization and magnetostriction of NiFe and NiFeRh films. J. Phys. Colloques 49, C8-1709–C8-1710 (1988).

[46] P. E. Mijnarends, S. Sahrakorpi, M. Lindroos, A. Bansil, Angle-resolved photoemission spectra, electronic structure, and spin-dependent scattering in Ni_1–x_Fe_x_ Permalloys. Phys. Rev. B 65, 075106 (2002).

[47] T. Neuman, D. S. Wang, P. Narang, Nanomagnonic cavities for strong spin-magnon coupling and magnon-mediated spin-spin interactions. Phys. Rev. Lett. 125, 247702 (2020). 33412028 10.1103/PhysRevLett.125.247702

[48] D. S. Wang, T. Neuman, P. Narang, Spin emitters beyond the point dipole approximation in nanomagnonic cavities. J. Phys. Chem. C 125, 6222–6228 (2021).

[49] M. A. Gilleo, S. Geller, Magnetic and crystallographic properties of substituted yttrium-iron garnet, 3Y_2_O_3_ *x*M_2_O_3_ (5–*x*)Fe_2_O_3_. Phys. Rev. 110, 73–78 (1958).

[50] L. Rondin, G. Dantelle, A. Slablab, F. Grosshans, F. Treussart, P. Bergonzo, S. Perruchas, T. Gacoin, M. Chaigneau, H.-C. Chang, V. Jacques, J.-F. Roch, Surface-induced charge state conversion of nitrogen-vacancy defects in nanodiamonds. Phys. Rev. B 82, 115449 (2010).

[51] N. P. de Leon, K. M. Itoh, D. Kim, K. K. Mehta, T. E. Northup, H. Paik, B. S. Palmer, N. Samarth, S. Sangtawesin, D. W. Steuerman, Materials challenges and opportunities for quantum computing hardware. Science 372, eabb2823 (2021). 33859004 10.1126/science.abb2823

[52] M. Sarovar, T. Proctor, K. Rudinger, K. Young, E. Nielsen, R. Blume-Kohout, Detecting crosstalk errors in quantum information processors. Quantum 4, 321 (2020). 10.1038/s41467-020-19074-4 PMC7588494 33106482

[53] T. Kimoto, J. A. Cooper, *Fundamentals of Silicon Carbide Technology: Growth, Characterization, Devices and Applications* (John Wiley & Sons, 2014).

[54] S. Castelletto, A. Boretti, Silicon carbide color centers for quantum applications. J. Phys. Photonics 2, 022001 (2020).

[55] S. Mathuna, T. O’Donnell, N. Wang, K. Rinne, Magnetics on silicon: An enabling technology for power supply on chip. IEEE Trans. Power Electron. 20, 585–592 (2005).

[56] Y. Zhao, Q. Song, S.-H. Yang, T. Su, W. Yuan, S. S. P. Parkin, J. Shi, W. Han, Experimental investigation of temperature-dependent gilbert damping in permalloy thin films. Sci. Rep. 6, 22890 (2016). 26961411 10.1038/srep22890 PMC4785429

[57] H. Y. Yuan, Y. Cao, A. Kamra, R. A. Duine, P. Yan, Quantum magnonics: When magnon spintronics meets quantum information science. Phys. Rep. 965, 1–74 (2022).

[58] M. Elyasi, Y. M. Blanter, G. E. W. Bauer, Resources of nonlinear cavity magnonics for quantum information. Phys. Rev. B 101, 054402 (2020).

[59] L.-A. Wu, H. J. Kimble, J. L. Hall, H. Wu, Generation of squeezed states by parametric down conversion. Phys. Rev. Lett. 57, 2520–2523 (1986). 10033788 10.1103/PhysRevLett.57.2520

[60] C. Leroux, L. C. G. Govia, A. A. Clerk, Enhancing cavity quantum electrodynamics via antisqueezing: Synthetic ultrastrong coupling. Phys. Rev. Lett. 120, 093602 (2018). 29547301 10.1103/PhysRevLett.120.093602

[61] W. Qin, A. Miranowicz, P.-B. Li, X.-Y. Lü, J. Q. You, F. Nori, Exponentially enhanced light-matter interaction, cooperativities, and steady-state entanglement using parametric amplification. Phys. Rev. Lett. 120, 093601 (2018). 29547303 10.1103/PhysRevLett.120.093601

[62] T. Sebastian, K. Schultheiss, B. Obry, B. Hillebrands, H. Schultheiss, Micro-focused brillouin light scattering: Imaging spin waves at the nanoscale. Front. Phys. 3, 10.3389/fphy.2015.00035 (2015).

[63] A. Vansteenkiste, J. Leliaert, M. Dvornik, M. Helsen, F. Garcia-Sanchez, B. Van Waeyenberge, The design and verification of mumax3. AIP Adv. 4, 107133 (2014).

[64] B. A. Ivanov, G. M. Wysin, Magnon modes for a circular two-dimensional easy-plane ferromagnet in the cone state. Phys. Rev. B 65, 134434 (2002).

[65] L. Körber, “Theory and simulation on nonlinear spin-wave dynamics in magnetic vortices,” thesis, Technische Universität Dresden, Dresden (2019).

[66] R. Nagy, M. Widmann, M. Niethammer, D. B. R. Dasari, I. Gerhardt, O. O. Soykal, M. Radulaski, T. Ohshima, J. Vučković, N. T. Son, I. G. Ivanov, S. E. Economou, C. Bonato, S.-Y. Lee, J. Wrachtrup, Quantum properties of dichroic silicon vacancies in silicon carbide. Phys. Rev. Applied 9, 034022 (2018).

[67] R. Verba, V. Tiberkevich, A. Slavin, Damping of linear spin-wave modes in magnetic nanostructures: Local, nonlocal, and coordinate-dependent damping. Phys. Rev. B 98, 104408 (2018).

