# Supplementary material for: Parametric magnon transduction to spin qubits
Source: Sci Adv. 2024 Mar 20;10(12):eadi2042. doi: 10.1126/sciadv.adi2042 (PMC10954226; doi:10.1126/sciadv.adi2042)
Supplement: Supplementary file 1 — Sections S1 to S5 Figs. S1 to S7 References [file sciadv.adi2042_sm.pdf]

Science Advances
AAAS

Supplementary Materials for

Parametric magnon transduction to spin qubits

Mauricio Bejarano *et al.*

Corresponding author: Helmut Schultheiss, h.schultheiss@hzdr.de

Sci. Adv. **10**, eadi2042 (2024)
DOI: 10.1126/sciadv.adi2042

This PDF file includes:

Sections S1 to S5
Figs. S1 to S7
References

section S1. Influence of the disc's dimension in the three-magnon splitting process

The magnon dispersion relation of the vortex disc is characterized by a discrete set of radial and azimuthal mode indices (*n,m*) resulting from the boundary conditions imposed in the corresponding direction. Given this discretization of the magnon spectra is a geometrical effect, by changing the dimensions of the disc the magnon dispersion relation changes accordingly. In Fig. S3 we compare the dispersion relation of the vortex magnons for discs with different diameters. The magnon dispersion relation changes in two major ways: first, with decreasing disc diameter the frequencies of the vortex modes increase, due to the stronger confinement of the modes in a smaller potential well. Second, with increasing disc diameter, the minima of each branch of the dispersion relation shifts towards higher azimuthal *m* indices. This shift occurs because it is energetically more favorable to redistribute the magnon intensity towards the perimeter of the disc by having higher azimuthal indices. Therefore, by changing the magnon dispersion relation, the disc's dimension determines which vortex magnons are involved in the three-magnon splitting process discussed in the main text.

section S2. Linear vortex dynamics

In this section, we provide a lengthier explanation of the diagonal ODMR intensity shown in Fig. 2B. Using our previous results published in *(30)*, in the main text we argue that the increasing ODMR intensity as the excitation frequency increases is because the vortex magnetization is more resonantly excited as the excitation frequency increases. To elaborate on this, in Fig. S4 we show a μBLS spectra for increasing excitation frequencies at 1 mW of microwave power, well below the three-magnon splitting threshold. We focus on this microwave power to obtain better visibility into the linearly-excited vortex dynamics. The regions with the highest BLS intensities in Fig. S4 correspond to the resonant excitation of the vortex eigenmodes with *m*=0 (due to the symmetry of our microwave antenna). This corresponds to the radial mode of the lowest 4 branches of the dispersion relation shown in Fig. S3B. However, we also observe faint, non-zero BLS intensities along the diagonal, at excitation frequencies that do not exactly match the resonant frequencies of the system, i.e., in the gaps between the dispersion branches of Fig. S3B and below 5.5 GHz. This lower-intensity BLS signal corresponds to a forced excitation of the magnetization. As the excitation frequency increases and approaches an eigenfrequency of the system, the BLS intensity gradually increases too, indicating larger magnon amplitudes, which in turn, results in larger dipolar fields. The gradually stronger dipolar fields add up to the antenna fields, resulting in overall larger microwave driving fields that increase the ODMR intensity in Fig. 2B.

section S3. μBLS microscopy on three-magnon splitting

Here we complement the micromagnetic simulations shown in Fig. 1E in the main text by discussing μBLS microscopy studies showing the frequency and field dependences of the three-magnon splitting process in the vortex disc. In Fig. S4A we show the detected μBLS frequencies in the disc as we increase the microwave excitation frequency at an out-of-plane magnetic field |**B**| = 99 mT. The continuous diagonal intensity that shifts linearly with increasing microwave frequency corresponds to the pump magnon mode f_0 which is directly driven by the antenna microwaves. Starting around $f_{\text{exc}} \approx$ 5.7 GHz two off-diagonal intensities appear, approximately

at a symmetric distance from $f_0/2$. These resonances correspond to the two secondary magnon modes, f_+ and f_-, that are generated during the three-magnon splitting process.

We now gradually change the external magnetic field to elucidate any field dependency impacting the three-magnon splitting in the disc. In Fig. S4B we show the μBLS spectra obtained for $f_{\text{exc}} = 6.1$ GHz as we increase the out-of-plane magnetic field. The most notorious consequence of increasing the field is the apparent branching and gradual suppression of the secondary modes that start around $|\mathbf{B}| = 100$ mT. This has already been reported and is still a subject of ongoing research. The most probable cause points to a change in the resonant magnon channels due to a topological effect that lifts the degeneracy of the secondary magnon doublets (*65*). While this is an interesting behavior, it is out of the scope of this work and thus it will not be discussed any further. Most importantly, we note that within the magnetic field range utilized in our experiments, the secondary modes are not completely suppressed. We also emphasize that the observed branching in Fig. S4B shows good agreement with the micromagnetic simulations in Fig. 1E in the main text. Moreover, the observed off-diagonal ODMR resonances around $f_{\text{exc}} = 2.5$ GHz and $f_{\text{exc}} = 3.25$ GHz in Fig. 2B of the main text match the resonant frequencies of the secondary magnons shown in Fig. S4B.

section S4. Power dependence of V_{Si}(V2) spin resonances

In this section, we discuss the typical microwave power dependence of ODMR spectra of V_{Si}(V2) when driven by antenna microwaves. Fig. S3A shows ODMR spectra for increasing microwave excitation powers for V_{Si}(V2) lying below the on-chip antenna (see inset). At this measurement position, the V_{Si}(V2) spin transitions are driven more efficiently given the antenna microwaves are perpendicular to the spin quantization axis (in this case, the *c*-axis) of the V_{Si}(V2). In Fig. S3A we observe the characteristic V_{Si}(V2) resonances ν_1 and ν_2, associated with $\Delta m_s = \pm 1$, increasing in linewidth as the microwave power increases. This power dependence is well known from standard ESR models. However, we observe an additional feature with negative ODMR contrast appearing at $f_{\text{exc}} = \gamma|\mathbf{B}|$ as the microwave power increases. We associate this feature with the presence of the hexagonal V_{Si} defect, known in literature as the V_{Si}(V1) (*66*). As the microwave power increases even further, additional resonances related to two-photon processes start to appear (*31*). In Fig. S3B we show individual ODMR spectra at three distinct microwave excitation powers where the three main resonances can be distinguished.

Notably, the ODMR spectrum in Fig. S3A is continuous as the microwave power increases, whereas the spectrum in Fig. 3A in the main text has a threshold-like activation of PL. We attribute this to the parametric generation of the magnon that is resonant to the V_{Si}(V2) defects. Only after the critical threshold amplitude of the pump magnon has been overcome, does the resonant magnon (at $|\mathbf{B}| = 99$ mT it is the f_- mode) appear, which then resonantly drives V_{Si}(V2) spin transitions. In the case of an antenna-generated driving field, the resonant excitation is continuous, which results in a continuous spectrum as the microwave excitation power increases.

section S5. Coupling the parametric magnons to single spins

In the main text we discuss the expected spin-magnon coupling strength with the parametric magnon modes. In this section, we discuss how these coupling strengths can be harnessed to

engineer hybrid magnon-quantum systems in the strong coupling regime, i.e., capable of transferring information in a coherent manner.

A useful parameter to characterize the strong coupling regime is the cooperativity C, which can be defined according to *(44)* as follows:

$$C = \frac{4 * g^2}{n * \kappa_m * \kappa_q}$$

with g as the spin-magnon coupling strength; $\kappa_m = \alpha_G \epsilon \omega$ as the intrinsic magnon damping rate, with α_G the Gilbert damping coefficient and ϵ the ellipticity coefficient of the magnon mode *(65)*; $\kappa_q = 1/T_2^*$ as the spin decoherence rate, with T_2^* as the spin coherence time; and $n = 1/\left(e^{\hbar\omega/k_B T} - 1\right)$ as the average number of thermal magnons. The strong coupling regime is achieved when $C > 1$ and $g > \kappa_m, \kappa_q$. We calculate the damping rates of the parametric magnons using the room-temperature Gilbert damping of permalloy $\alpha_G = 0.007$ and the ellipticity coefficients from *(67)* $\epsilon_- = 5.1$ and $\epsilon_+ = 3$. This results in $\kappa_{m,-} = 2\pi \times 89$ MHz for the f_- mode and $\kappa_{m,+} = 2\pi \times 73$ MHz for the f_+ mode. From the ODMR linewidth at low microwave powers (Fig. S6) we estimate a spin ensemble decoherence rate of $\kappa_q = 15$ MHz ($T_2^* = 67$ ns). As explained in the main text, given the coupling strengths are not sufficient to overcome the intrinsic decoherence processes in both the magnonic and quantum subsystems, here we explore the outlook of coupling the parametric magnons to single V_{Si}(V2) spins, which have a much smaller decoherence rate of 10 kHz, stemming from a larger $T_2^* = 100$ µs *(32)*. In Fig. S7 we show the calculated room-temperature cooperativities of the hybrid parametric magnon-single spin system. Remarkably, despite the large magnon damping rates, the coupling strengths from the parametric magnons result in cooperativities C>1 in multiple regions below the disc when coupling to single spins. Due to the large spin density of permalloy, cooperativities C>1 can be obtained as far away as 200 nm from the disc (in the case of the f_+ mode). At a distance of 25 nm away from the disc, the cooperativities are C_-=23 and C_+=138 for the f_- and f_+ mode respectively. For comparison purposes, this is up to 8 times the cooperativities obtained in single NV/YIG systems at comparable distances *(47,48)*. To note, the coupling with the f_+ mode yields larger cooperativities than the f_- mode given the former has a lower damping rate as mentioned before.

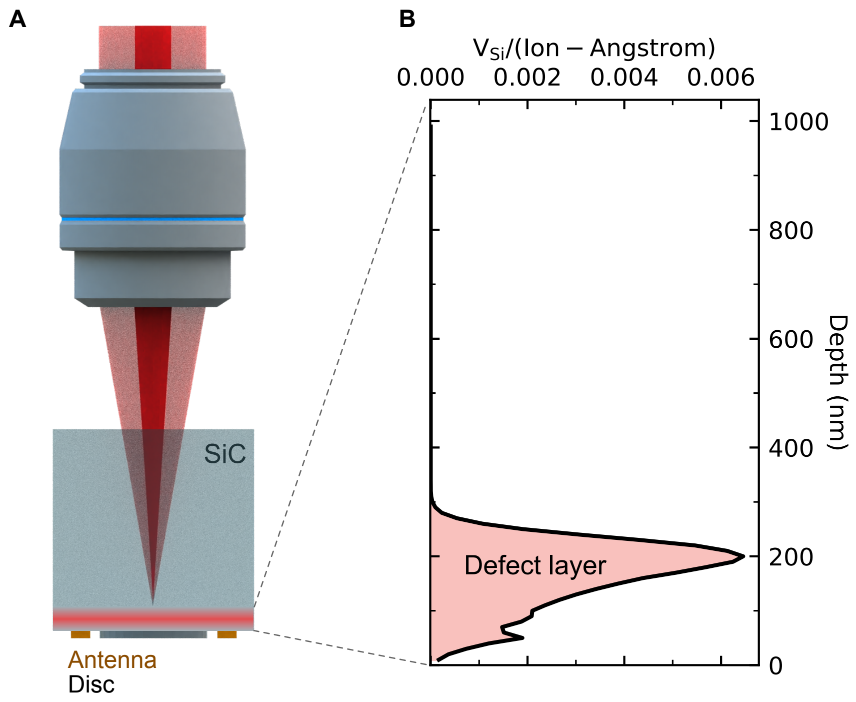

Fig. S1. **Depth profile of V_{Si} defects.** (A) Experimental geometry for probing V_{Si}. The 785 nm excitation laser is focused with a microscope objective onto the defect-rich layer through the backside of the SiC sample. The emitted PL is collected through the same objective. (B) Simulated V_{Si} concentration through the depth of the substrate.

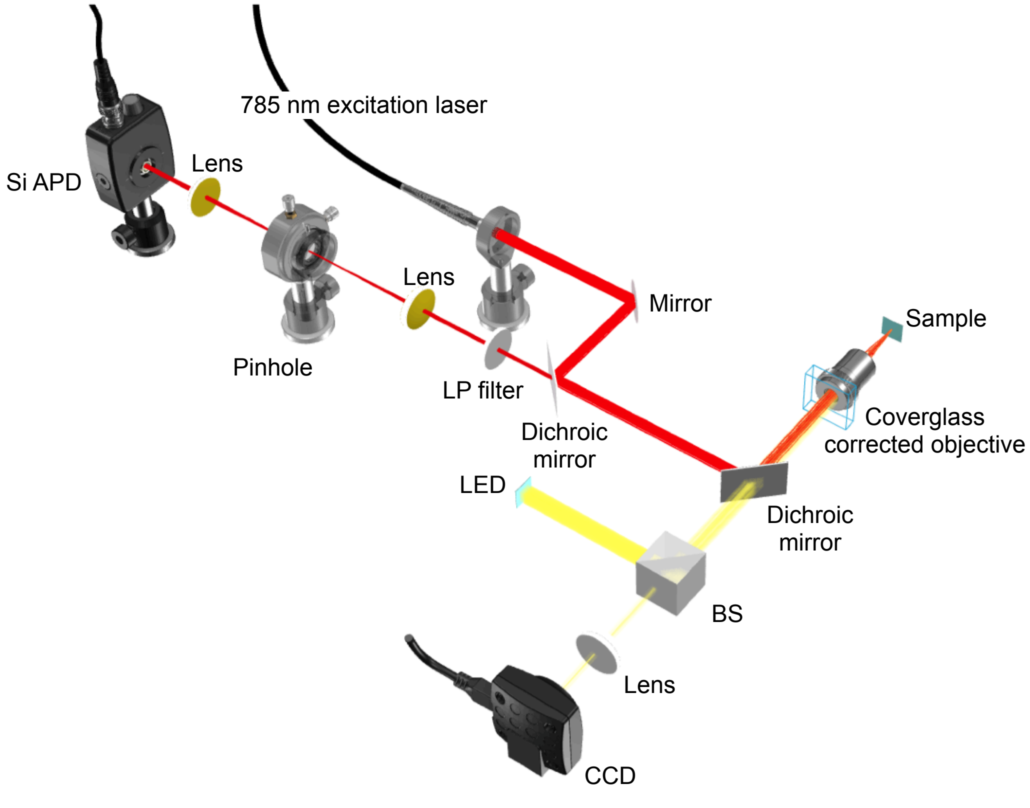

Fig. S2. **Schematic of the experimental setup for ODMR experiments.** APD: avalanche photodetector, LP: longpass, LED: light-emitting diode, BS: beamsplitter, CCD: charge-coupled device.

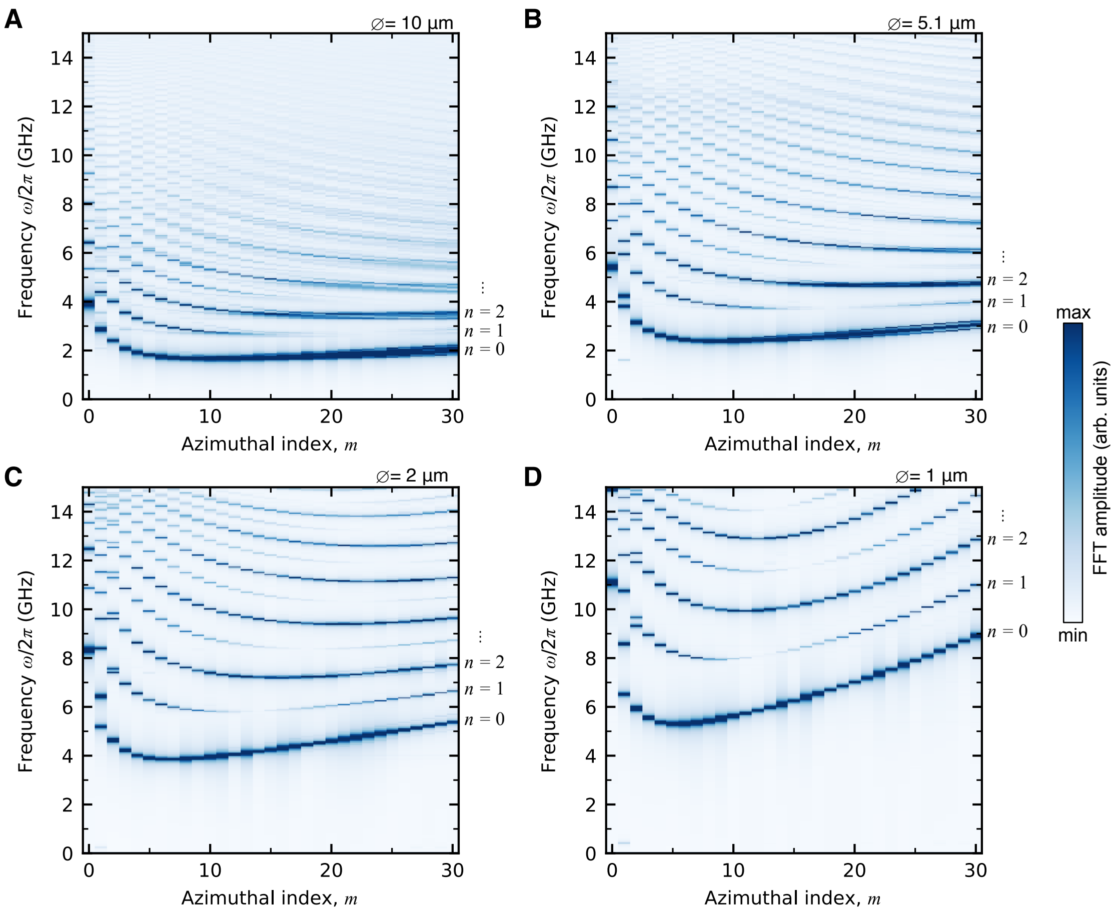

Fig. S3. **Dispersion relation of the vortex magnons.** (A) For a disc diameter of 10 µm, (B) 5.1 µm, (C) 2 µm, and (D) 1 µm. Data taken from *(65)*, obtained with micromagnetic simulations and provided by the author.

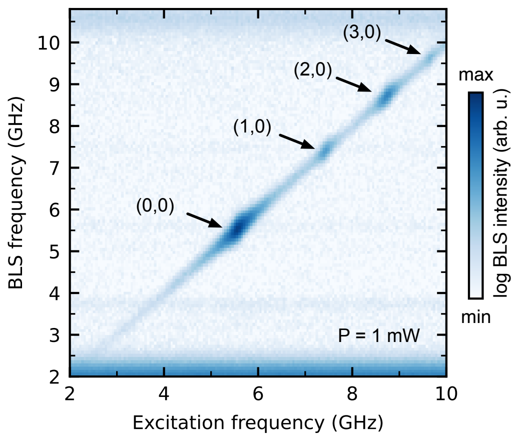

Fig. S4. **Magnon excitation spectra below the 3MS threshold.** The four regions with largest BLS intensities correspond to the directly excited vortex modes from the lowest four branches of the magnon dispersion relation. Color scale is logarithmic. Data replotted from our earlier work in *(30)*.

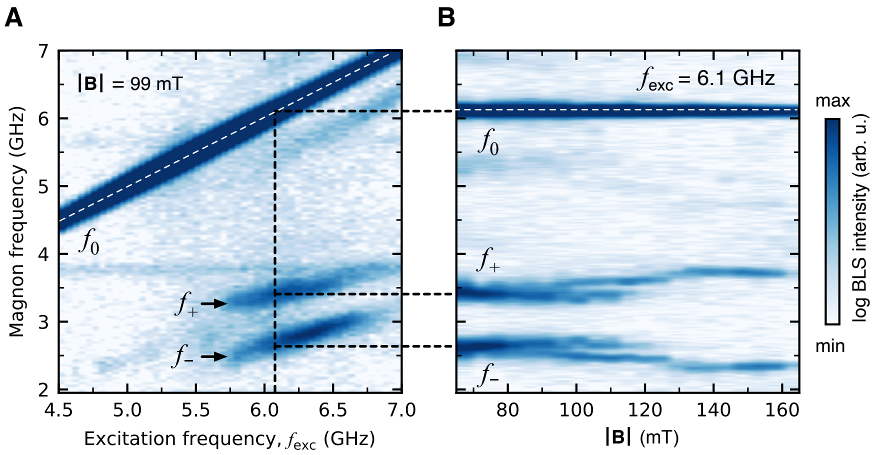

Fig. S5. **Magnon excitation spectra above the 3MS threshold.** (A) µBLS spectra for increasing microwave excitation frequencies at |**B**| = 99 mT. (B) µBLS spectra for increasing out-of-plane magnetic fields at $f_{\mathrm{exc}} =$ 6.1 GHz. Both spectra in (A) and (B) were obtained with a microwave power of 21 dBm.

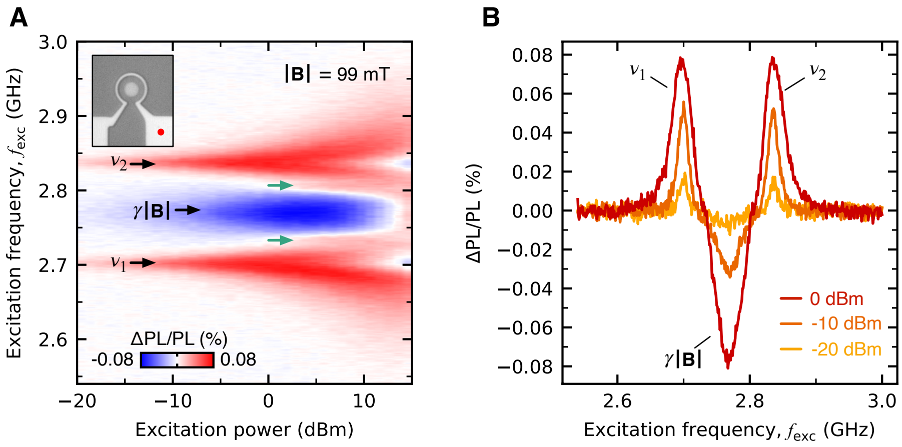

Fig. S6. **Power dependence of V_{Si}(V2) spin resonances.** (A) ODMR spectrum at increasing microwave excitation powers at |**B**| = 99 mT. Inset shows the measurement position below the microwave antenna. ν_1 and ν_2 correspond to the V_{Si}(V2) resonances and γ|**B**| corresponds to the V_{Si}(V1) resonance. Green arrows indicate two-photon transitions. (B) ODMR spectra from (A) at three distinct microwave excitation powers.

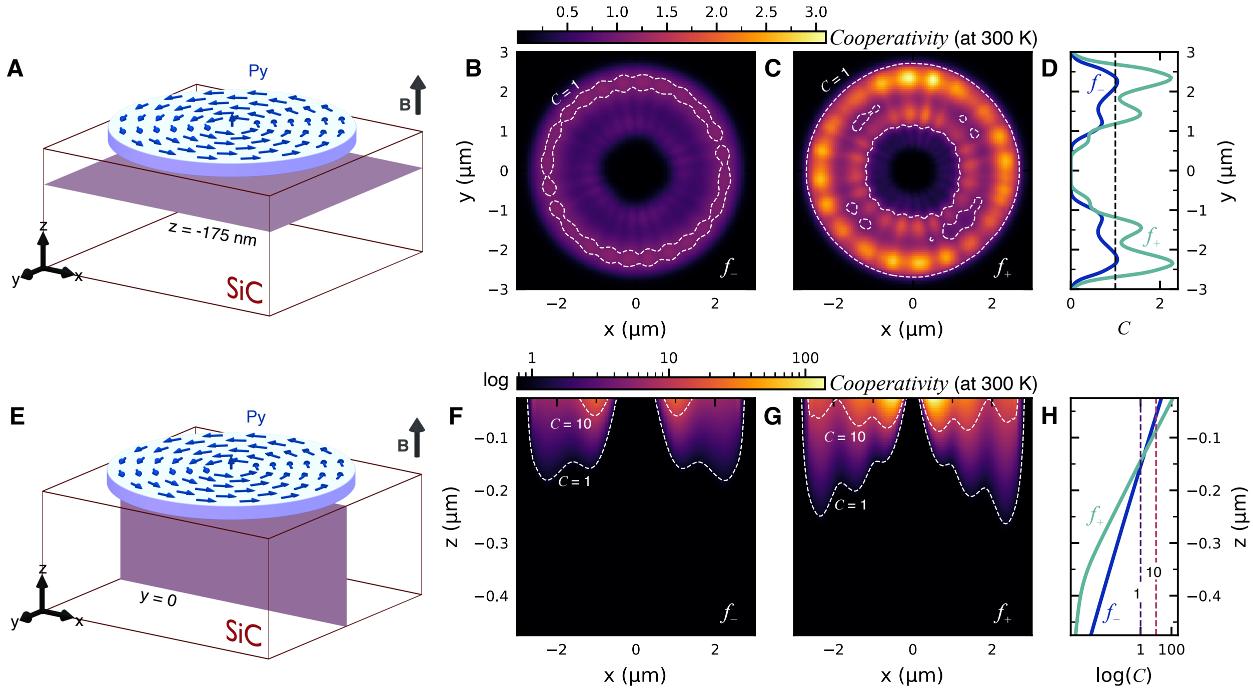

Fig. S7. **Room-temperature cooperativity between the vortex magnons and a single V_{Si}(V2).** (A) Spatial cooperativity at a plane located 175 nm below the disc. The surface of the SiC substrate is at z = 0 nm. (B) and (C) show the cooperativities for the parametric magnons f_- and f_+ at $|\mathbf{B}|$ = 90 mT and $|\mathbf{B}|$ = 130 mT respectively at the plane shown in (A). (D) Cooperativities along x = 0 µm from the intensity maps shown in (B) and (C). Dashed line is the C = 1 minimum. (E) Spatial cooperativity at a cross-section plane located at y = 0 µm starting 25 nm below the disc. (F) and (G) show the cooperativities in logarithmic scale for the parametric magnons f_- and f_+ at $|\mathbf{B}|$ = 90 mT and $|\mathbf{B}|$ = 130 mT respectively at the plane shown in (E). (H) Cooperativities for the f_- and f_+ modes along x = −1.05 µm and x = 0.6 µm as extracted from (F) and (G) respectively.

REFERENCES

1. R. P. Feynman, Simulating physics with computers. *Int. J. Theor. Phys.* **21**, 467–488 (1982).
2. J. I. Cirac, P. Zoller, Quantum computations with cold trapped ions. *Phys. Rev. Lett.* **74**, 4091–4094 (1995).
3. D. Castelvecchi, IBM releases first-ever 1,000-qubit quantum chip. *Nature* **624**, 238–238 (2023).
4. Y. Nakamura, Y. A. Pashkin, J. S. Tsai, Coherent control of macroscopic quantum states in a single-Cooper-pair box. *Nature* **398**, 786–788 (1999).
5. C. D. Bruzewicz, J. Chiaverini, R. McConnell, J. M. Sage, Trapped-ion quantum computing: Progress and challenges. *Appl. Phys. Rev.* **6**, 021314 (2019).
6. F. Jelezko, J. Wrachtrup, Single defect centres in diamond: A review. *Phys. Status Solidi A* **203**, 3207–3225 (2006).
7. P. G. Baranov, A. P. Bundakova, A. A. Soltamova, S. B. Orlinskii, I. V. Borovykh, R. Zondervan, R. Verberk, J. Schmidt, Silicon vacancy in SiC as a promising quantum system for single-defect and single-photon spectroscopy. *Phys. Rev. B* **83**, 125203 (2011).
8. D. Loss, D. P. DiVincenzo, Quantum computation with quantum dots. *Phys. Rev. A* **57**, 120–126 (1998).
9. D. S. Wang, M. Haas, P. Narang, Quantum interfaces to the nanoscale. *ACS Nano* **15**, 7879–7888 (2021).
10. S. Pirandola, S. L. Braunstein, Physics: Unite to build a quantum Internet. *Nature* **532**, 169–171 (2016).
11. C. Monroe, R. Raussendorf, A. Ruthven, K. R. Brown, P. Maunz, L.-M. Duan, J. Kim, Large-scale modular quantum-computer architecture with atomic memory and photonic interconnects. *Phys. Rev. A* **89**, 022317 (2014).
12. Z.-L. Xiang, S. Ashhab, J. Q. You, F. Nori, Hybrid quantum circuits: Superconducting circuits interacting with other quantum systems. *Rev. Mod. Phys.* **85**, 623–653 (2013).
13. N. Lauk, N. Sinclair, S. Barzanjeh, J. P. Covey, M. Saffman, M. Spiropulu, C. Simon, Perspectives on quantum transduction. *Quantum Sci. Technol.* **5**, 020501 (2020).
14. D. D. Awschalom, C. R. Du, R. He, F. J. Heremans, A. Hoffmann, J. Hou, H. Kurebayashi, Y. Li, L. Liu, V. Novosad, J. Sklenar, S. E. Sullivan, D. Sun, H. Tang, V. Tyberkevych, C. Trevillian, A. W. Tsen, L. R. Weiss, W. Zhang, X. Zhang, L. Zhao, Ch. W. Zollitsch, Quantum engineering with hybrid magnonic systems and materials (*Invited Paper*). *IEEE Trans. Quantum Eng.* **2**, 1–36 (2021).
15. Y. Li, W. Zhang, V. Tyberkevych, W.-K. Kwok, A. Hoffmann, V. Novosad, Hybrid magnonics: Physics, circuits, and applications for coherent information processing. *J. Appl. Phys.* **128**, 130902 (2020).

16. D. Lachance-Quirion, Y. Tabuchi, A. Gloppe, K. Usami, Y. Nakamura, Hybrid quantum systems based on magnonics. *Appl. Phys. Express* **12**, 070101 (2019).
17. D. Lachance-Quirion, Y. Tabuchi, S. Ishino, A. Noguchi, T. Ishikawa, R. Yamazaki, Y. Nakamura, Resolving quanta of collective spin excitations in a millimeter-sized ferromagnet. *Sci. Adv.* **3**, e1603150 (2017).
18. D. Lachance-Quirion, S. P. Wolski, Y. Tabuchi, S. Kono, K. Usami, Y. Nakamura, Entanglement-based single-shot detection of a single magnon with a superconducting qubit. *Science* **367**, 425–428 (2020).
19. Y. Tabuchi, S. Ishino, A. Noguchi, T. Ishikawa, R. Yamazaki, K. Usami, Y. Nakamura, Coherent coupling between a ferromagnetic magnon and a superconducting qubit. *Science* **349**, 405–408 (2015).
20. P. Andrich, C. F. de las Casas, X. Liu, H. L. Bretscher, J. R. Berman, F. J. Heremans, P. F. Nealey, D. D. Awschalom, Long-range spin wave mediated control of defect qubits in nanodiamonds. *Npj Quantum Inf.* **3**, 28 (2017).
21. T. van der Sar, F. Casola, R. Walsworth, A. Yacoby, Nanometre-scale probing of spin waves using single electron spins. *Nat. Commun.* **6**, 7886 (2015).
22. F. Casola, T. van der Sar, A. Yacoby, Probing condensed matter physics with magnetometry based on nitrogen-vacancy centres in diamond. *Nat. Rev. Mater.* **3**, 17088 (2018).
23. M. Fukami, D. R. Candido, D. D. Awschalom, M. E. Flatté, Opportunities for long-range magnon-mediated entanglement of spin qubits via on- and off-resonant coupling. *PRX Quantum* **2**, 040314 (2021).
24. Y. Li, V. G. Yefremenko, M. Lisovenko, C. Trevillian, T. Polakovic, T. W. Cecil, P. S. Barry, J. Pearson, R. Divan, V. Tyberkevych, C. L. Chang, U. Welp, W.-K. Kwok, V. Novosad, Coherent coupling of two remote magnonic resonators mediated by superconducting circuits. *Phys. Rev. Lett.* **128**, 047701 (2022).
25. S. Kosen, A. F. van Loo, D. A. Bozhko, L. Mihalceanu, A. D. Karenowska, Microwave magnon damping in YIG films at millikelvin temperatures. *APL Mater.* **7**, 101120 (2019).
26. T. Shinjo, T. Okuno, R. Hassdorf, K. Shigeto, T. Ono, Magnetic vortex core observation in circular dots of permalloy. *Science* **289**, 930–932 (2000).
27. M. Buess, R. Höllinger, T. Haug, K. Perzlmaier, U. Krey, D. Pescia, M. R. Scheinfein, D. Weiss, C. H. Back, Fourier transform imaging of spin vortex eigenmodes. *Phys. Rev. Lett.* **93**, 077207 (2004).
28. M. Buess, T. P. J. Knowles, R. Höllinger, T. Haug, U. Krey, D. Weiss, D. Pescia, M. R. Scheinfein, C. H. Back, Excitations with negative dispersion in a spin vortex. *Phys. Rev. B* **71**, 104415 (2005).

29. B. Ivanov, C. Zaspel, High frequency modes in vortex-state nanomagnets. *Phys. Rev. Lett.* **94**, 027205 (2005).
30. K. Schultheiss, R. Verba, F. Wehrmann, K. Wagner, L. Körber, T. Hula, T. Hache, A. Kákay, A. A. Awad, V. Tiberkevich, A. N. Slavin, J. Fassbender, H. Schultheiss, Excitation of whispering gallery magnons in a magnetic vortex. *Phys. Rev. Lett.* **122**, 097202 (2019).
31. H. Kraus, V. A. Soltamov, D. Riedel, S. Väth, F. Fuchs, A. Sperlich, P. G. Baranov, V. Dyakonov, G. V. Astakhov, Room-temperature quantum microwave emitters based on spin defects in silicon carbide. *Nat. Phys.* **10**, 157–162 (2014).
32. M. Widmann, S.-Y. Lee, T. Rendler, N. T. Son, H. Fedder, S. Paik, L.-P. Yang, N. Zhao, S. Yang, I. Booker, A. Denisenko, M. Jamali, S. A. Momenzadeh, I. Gerhardt, T. Ohshima, A. Gali, E. Janzén, J. Wrachtrup, Coherent control of single spins in silicon carbide at room temperature. *Nat. Mater.* **14**, 164–168 (2015).
33. E. Sörman, N. T. Son, W. M. Chen, O. Kordina, C. Hallin, E. Janzén, Silicon vacancy related defect in 4H and 6H SiC. *Phys. Rev. B* **61**, 2613–2620 (2000).
34. E. Janzén, A. Gali, P. Carlsson, A. Gällström, B. Magnusson, N. T. Son, The silicon vacancy in SiC. *Phys. B Condens. Matter* **404**, 4354–4358 (2009).
35. N. Mizuochi, S. Yamasaki, H. Takizawa, N. Morishita, T. Ohshima, H. Itoh, J. Isoya, Continuous-wave and pulsed EPR study of the negatively charged silicon vacancy with S = 3/2 and C_{3v} symmetry in n -type $4H-$ SiC. *Phys. Rev. B* **66**, 235202 (2002).
36. S. A. Tarasenko, A. V. Poshakinskiy, D. Simin, V. A. Soltamov, E. N. Mokhov, P. G. Baranov, V. Dyakonov, G. V. Astakhov, Spin and optical properties of silicon vacancies in silicon carbide—A review. *Phys. Status Solidi B* **255**, 1700258 (2018).
37. D. Simin, F. Fuchs, H. Kraus, A. Sperlich, P. G. Baranov, G. V. Astakhov, V. Dyakonov, High-precision angle-resolved magnetometry with uniaxial quantum centers in silicon carbide. *Phys. Rev. Applied* **4**, 014009 (2015).
38. V. S. L'vov, Nonlinear dynamics and kinetics of magnons, in *Nonlinear Waves 3,* A. V. Gaponov-Grekhov, M. I. Rabinovich, J. Engelbrecht, Eds. (Springer Berlin Heidelberg, 1990), pp. 224–239.
39. H. Suhl, The theory of ferromagnetic resonance at high signal powers. *J. Phys. Chem. Solid* **1**, 209–227 (1957).

40. R. E. Camley, Three-magnon processes in magnetic nanoelements: Quantization and localized mode effects. *Phys. Rev. B* **89**, 214402 (2014).

41. R. Verba, L. Körber, K. Schultheiss, H. Schultheiss, V. Tiberkevich, A. Slavin, Theory of three-magnon interaction in a vortex-state magnetic nanodot. *Phys. Rev. B* **103**, 014413 (2021).

42. V. L'vov, *Wave Turbulence under Parametric Excitation: Applications to Magnets*, Springer Series in Nonlinear Dynamics (Springer Berlin Heidelberg, 2012).

43. D. W. Vernooy, A. Furusawa, N. Ph. Georgiades, V. S. Ilchenko, H. J. Kimble, Cavity QED with high-*Q* whispering gallery modes. *Phys. Rev. A* **57**, R2293–R2296 (1998).

44. D. R. Candido, G. D. Fuchs, E. Johnston-Halperin, M. E. Flatté, Predicted strong coupling of solid-state spins via a single magnon mode. *Mater. Quantum Technol.* **1**, 011001 (2021).

45. K. Ounadjela, H. Lefakis, V. S. Speriosu, C. Hwang, P. S. Alexopoulos, Thickness dependence of magnetization and magnetostriction of NiFe and NiFeRh films. *J. Phys. Colloques* **49**, C8-1709–C8-1710 (1988).

46. P. E. Mijnarends, S. Sahrakorpi, M. Lindroos, A. Bansil, Angle-resolved photoemission spectra, electronic structure, and spin-dependent scattering in $Ni_{1-x}Fe_x$ Permalloys. *Phys. Rev. B* **65**, 075106 (2002).

47. T. Neuman, D. S. Wang, P. Narang, Nanomagnonic cavities for strong spin-magnon coupling and magnon-mediated spin-spin interactions. *Phys. Rev. Lett.* **125**, 247702 (2020).

48. D. S. Wang, T. Neuman, P. Narang, Spin emitters beyond the point dipole approximation in nanomagnonic cavities. *J. Phys. Chem. C* **125**, 6222–6228 (2021).

49. M. A. Gilleo, S. Geller, Magnetic and crystallographic properties of substituted yttrium-iron garnet, $3Y_2O_3\ xM_2O_3\ (5–x)Fe_2O_3$. *Phys. Rev.* **110**, 73–78 1958).

50. L. Rondin, G. Dantelle, A. Slablab, F. Grosshans, F. Treussart, P. Bergonzo, S. Perruchas, T. Gacoin, M. Chaigneau, H.-C. Chang, V. Jacques, J.-F. Roch, Surface-induced charge state conversion of nitrogen-vacancy defects in nanodiamonds. *Phys. Rev. B* **82**, 115449 (2010).

51. N. P. de Leon, K. M. Itoh, D. Kim, K. K. Mehta, T. E. Northup, H. Paik, B. S. Palmer, N. Samarth, S. Sangtawesin, D. W. Steuerman, Materials challenges and opportunities for quantum computing hardware. *Science* **372**, eabb2823 (2021).

52. M. Sarovar, T. Proctor, K. Rudinger, K. Young, E. Nielsen, R. Blume-Kohout, Detecting crosstalk errors in quantum information processors. *Quantum* **4**, 321 (2020).

53. T. Kimoto, J. A. Cooper, *Fundamentals of Silicon Carbide Technology: Growth, Characterization, Devices and Applications* (John Wiley & Sons, 2014).
54. S. Castelletto, A. Boretti, Silicon carbide color centers for quantum applications. *J. Phys. Photonics* **2**, 022001 (2020).
55. S. Mathuna, T. O'Donnell, N. Wang, K. Rinne, Magnetics on silicon: An enabling technology for power supply on chip. *IEEE Trans. Power Electron.* **20**, 585–592 (2005).
56. Y. Zhao, Q. Song, S.-H. Yang, T. Su, W. Yuan, S. S. P. Parkin, J. Shi, W. Han, Experimental investigation of temperature-dependent gilbert damping in permalloy thin films. *Sci. Rep.* **6**, 22890 (2016).
57. H. Y. Yuan, Y. Cao, A. Kamra, R. A. Duine, P. Yan, Quantum magnonics: When magnon spintronics meets quantum information science. *Phys. Rep.* **965**, 1–74 (2022).
58. M. Elyasi, Y. M. Blanter, G. E. W. Bauer, Resources of nonlinear cavity magnonics for quantum information. *Phys. Rev. B* **101**, 054402 (2020).
59. L.-A. Wu, H. J. Kimble, J. L. Hall, H. Wu, Generation of squeezed states by parametric down conversion. *Phys. Rev. Lett.* **57**, 2520–2523 (1986).
60. C. Leroux, L. C. G. Govia, A. A. Clerk, Enhancing cavity quantum electrodynamics via antisqueezing: Synthetic ultrastrong coupling. *Phys. Rev. Lett.* **120**, 093602 (2018).
61. W. Qin, A. Miranowicz, P.-B. Li, X.-Y. Lü, J. Q. You, F. Nori, Exponentially enhanced light-matter interaction, cooperativities, and steady-state entanglement using parametric amplification. *Phys. Rev. Lett.* **120**, 093601 (2018).
62. T. Sebastian, K. Schultheiss, B. Obry, B. Hillebrands, H. Schultheiss, Micro-focused brillouin light scattering: Imaging spin waves at the nanoscale. *Front. Phys.* **3**, 10.3389/fphy.2015.00035 (2015).
63. A. Vansteenkiste, J. Leliaert, M. Dvornik, M. Helsen, F. Garcia-Sanchez, B. Van Waeyenberge, The design and verification of mumax3. *AIP Adv.* **4**, 107133 (2014).
64. B. A. Ivanov, G. M. Wysin, Magnon modes for a circular two-dimensional easy-plane ferromagnet in the cone state. *Phys. Rev. B* **65**, 134434 (2002).
65. L. Körber, "Theory and simulation on nonlinear spin-wave dynamics in magnetic vortices," thesis, Technische Universität Dresden, Dresden (2019).
66. R. Nagy, M. Widmann, M. Niethammer, D. B. R. Dasari, I. Gerhardt, O. O. Soykal, M. Radulaski, T. Ohshima, J. Vučković, N. T. Son, I. G. Ivanov, S. E. Economou, C. Bonato, S.-Y. Lee, J. Wrachtrup, Quantum properties of dichroic silicon vacancies in silicon carbide. *Phys. Rev. Applied* **9**, 034022 (2018).

67. R. Verba, V. Tiberkevich, A. Slavin, Damping of linear spin-wave modes in magnetic nanostructures: Local, nonlocal, and coordinate-dependent damping. *Phys. Rev. B* **98**, 104408 (2018).
